# Patient satisfaction with mental and physical health services: Findings from a UK-wide online survey

**DOI:** 10.12688/wellcomeopenres.17973.1

**Published:** 2022-08-01

**Authors:** Elizabeth J. Kirkham, Sue Fletcher-Watson, Iona Beange, Stella W.Y. Chan, Stephen M. Lawrie

**Affiliations:** 1 Division of Psychiatry, Centre for Clinical Brain Sciences, University of Edinburgh, Edinburgh, UK; 2 School of Psychology and Clinical Language Sciences, University of Reading, Reading, UK

**Keywords:** patient satisfaction; mental health services; NHS; mental illness; psychiatry; therapy; antidepressants; physical health services; health services

## Abstract

**Introduction** - Despite extensive debate surrounding mental health services in the UK, there is little empirical evidence regarding the views of those who use them. We therefore used data collected as part of a wider survey to examine satisfaction amongst those seeking treatment from mental and physical health services.

**Methods** – An online survey designed with input from people with experience of mental illness was used to measure satisfaction with NHS mental and physical health services at first contact and in the previous 12 months.

**Results** – A total of 2187 people responded. During the 12 months prior to the survey, 526 respondents had sought mental health care and 1379 had sought physical health care. Participants were significantly more satisfied with their most recent contact with mental health services (48.1% very/satisfied) than with their first contact (38.2% very/satisfied). More than 1 in 10 respondents who sought mental health care (11.4%) stated that they received no treatment/support from the NHS, compared to approximately 1 in 20 respondents who sought physical health care (4.6%). Of those who received the mental health treatment they requested (n = 424), most were satisfied or very satisfied with their care (54.7%), although this was lower than the corresponding figure (77.9%) for satisfaction with physical health care received (n = 1190).

**Conclusion** –There was evidence that mental health services are satisfactory for a slim majority of users, but people were generally more satisfied with NHS physical health care. This survey was conducted in the year prior to the coronavirus disease 2019 pandemic. Future research could examine what influences satisfaction with care and whether this picture has changed following the emergence of the pandemic and consequent impact on health service delivery and daily life.

## Introduction

Collecting data on patient satisfaction with care and treatment is a key component of a responsive health service ( Mosler *et al.*, 2020; Werkkala *et al.*, 2020). Such measures of satisfaction are collected annually by the British Social Attitudes survey across England, Scotland and Wales ( Appleby *et al.*, 2020), and by the National Patient Experience surveys in England. However, despite the legal requirement to give “parity of esteem” to mental and physical health in the Health and Social Act 2012 ( Health and Social Care Act, 2012), there is a notable lack of published information about satisfaction with UK mental health services. For example, mental (ill) health is not explicitly discussed in the latest report on public satisfaction with health and social care ( Appleby *et al.*, 2020). Similarly, NHS England’s National Patient Experience surveys focus only on those who receive care from Community Mental Health services, and as such may miss the views of people who receive mental health care via other NHS routes, as well as those who have been unable to access NHS mental health care at all. Whilst some studies have assessed patient satisfaction with NHS mental health services, they tend to be specific to one locality (e.g. Mosler *et al.*, 2020) or treatment type (e.g. Ward *et al.*, 2013; Wykes *et al.*, 2018).

The absence of large-scale data focused on the views of people with mental illness further marginalises people who already face stigma within health care and beyond ( Clement *et al.*, 2015; Somerville, 2021). Without an overall picture of people’s satisfaction with mental health services, it is difficult to gauge how these services compare to physical health services, or whether satisfaction has changed over time, as knowledge of mental health has increased. The ability to make such comparisons would have multiple benefits, from identifying any mental health conditions which require additional provision ( Dale *et al.*, 2017; Parliamentary and Health Service Ombudsman, 2017), to contributing to ongoing debates about the orientation and role of psychiatry in treating mental health conditions ( Gardner & Kleinman, 2019).

Any research which examines satisfaction with NHS services must consider the context in which they are provided. NHS mental health services were under significant pressure even before the coronavirus disease 2019 (COVID-19) pandemic, with a lack of access to crisis care ( Care Quality Commission, 2020a), reductions in numbers of mental health doctors and nurses ( Trades Union Congress, 2018), and acute services operating beyond capacity ( Crisp *et al.*, 2016). In a recent citizen science project facilitated by one of our authors (IB; Depression Detectives, 2021), people with experience of depression highlighted long-standing difficulties in accessing appropriate mental health treatment from the NHS. This supports previous concerns that people whose needs fall between Improving Access to Psychological Therapies (IAPT) and secondary care are struggling to access treatment ( Martin *et al.*, 2022; Newbigging *et al.*, 2018), and anecdotal reports of patients turning to the private sector for specialist or long-term treatment.

It is therefore important to distinguish between people’s satisfaction with accessing mental health services in the UK, and satisfaction with the treatment they actually receive if and when they are able to access care. To do this we used NHS satisfaction data collected as part of a wider survey examining attitudes to sharing mental health data ( Kirkham *et al.*, 2022a) from two perspectives: satisfaction with *any contact* with NHS mental and physical health services (irrespective of whether or not treatment was received), and satisfaction with *treatment received from* NHS mental and physical health services. Satisfaction with contact was measured for both first contact and recent (12 month) contact, whilst satisfaction with treatment focused on the previous 12 months.

The objectives were to examine:

- participants’ satisfaction with the NHS response when they first sought mental vs. physical health care
- participants’ access to and satisfaction with their recent NHS treatment for mental vs. physical health conditions
- change in satisfaction with NHS mental health services over time
- satisfaction with NHS mental health services by type of treatment and mental health condition.

## Methods

### Ethics and consent

The research received ethical approval from the School of Health in Social Science Research Ethics Committee at the University of Edinburgh (REF: STAFF132, approval granted 13.12.2018). The authors assert that all procedures contributing to this work comply with the ethical standards of the relevant national and institutional committees on human experimentation and with the Helsinki Declaration of 1975, as revised in 2008. All participants provided written informed consent as indicated by selecting check boxes at the beginning of the online survey.

### Study design and population

A first draft of the survey was designed by the research team. This draft was presented to a group of people with lived experience of mental illness, and changes were made based on their feedback. The survey was also sent to research clinicians and further suggested alterations were incorporated. The whole survey covered participants’ views about sharing mental and physical health data, their personal experience of mental and physical health, their satisfaction with NHS mental and physical health services, and their demographic information. The survey was administered online through the Qualtrics platform and had a median response time of 11 minutes. The dataset and survey script are available as underlying and extended data, respectively ( Kirkham *et al.*, 2022b).

Participants were recruited from December 2018 to August 2019 via social media, posters, and in-person at a science festival. Several research and clinical teams around the UK promoted the survey through their networks, but there was no direct recruitment of patients via clinical settings. It was evident in the first month of data collection that, as with many questionnaire surveys, the sample was skewed towards white females with high levels of education. In light of this, efforts were made to capture a broader range of the UK population. Specifically, a Facebook advertising company was commissioned to advertise the survey to potential male participants, and to raise awareness of the survey amongst people who did not have degree-level qualifications. In addition, Prolific Academic was used to invite people from ethnic minorities to take part. As the survey was part of a fixed-term project to examine attitudes to mental health data, we did not have a target sample size. Rather, we stopped data collection once we had used the aforementioned routes to make the dataset as representative of the wider UK population as possible.

The inclusion criteria for the survey were that participants were age 16 or over and had used the NHS (for any reason and at any time). People with mental illness were deliberately over-sampled because this survey’s primary purpose was to find out the views of people with mental illness, a group of individuals whose opinions are often under-represented in research and wider society. Participants were classified as having experience of mental illness if they self-reported having had a mental illness at some time in their life (formal diagnosis was not required). In cases where participants stated that they had experienced multiple mental health conditions, they were also asked to choose a “primary” mental health condition which had the greatest impact on their life.

All participants took part voluntarily. The majority, including those recruited via Facebook, did not receive payment, though the 174 individuals who took part through Prolific Academic were paid approximately £2 each. A total of 2187 participants contributed data to the survey. The number of participants responding to each question varied, due to participant drop-out and built-in exclusion criteria (e.g. people were not asked specific questions about treatment for mental illness if they reported no experience of mental illness). Participants were only included in a given analysis if they provided a response to every question included in that analysis. The number of participants included in each analysis is indicated in the Results section. 

### Measurement of satisfaction with the NHS

**Figure 1.**
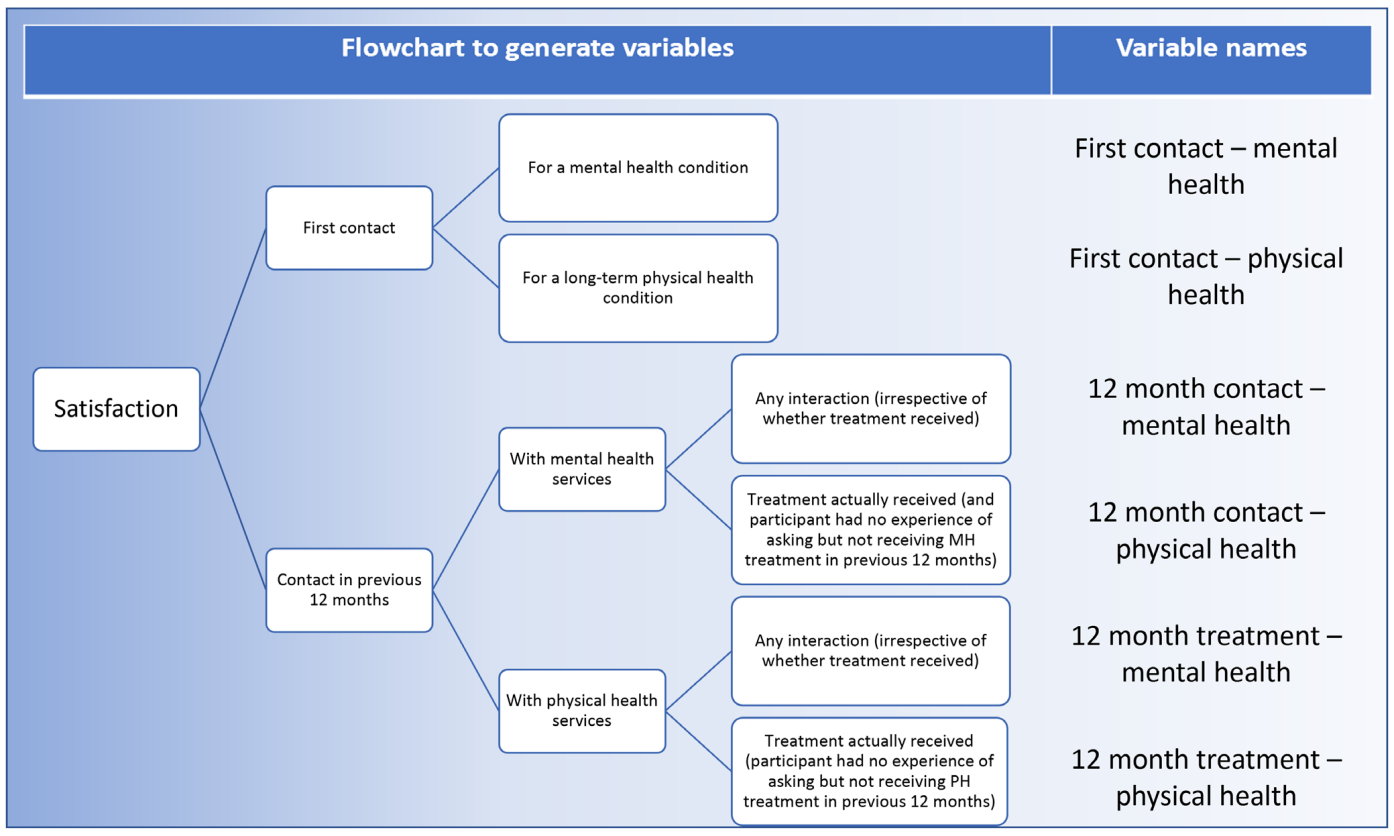
Creation of satisfaction variables for use in analysis.

The survey included four questions which focused on satisfaction with NHS services (extended data, Kirkham *et al.*, 2022b), with each item measured on a 5-point Likert scale from “very dissatisfied” to “very satisfied”. We used these questions to generate six variables to test our research questions ( Figure 1). These six variables covered satisfaction with the NHS [mental/physical] provision the first time an individual sought help for a health condition (“first contact”), how satisfied individuals were with the NHS mental/physical response provided in the 12 months prior to filling in the survey (“12 month contact”), and how satisfied they were with the actual treatment they were given in these 12 months (“12 month treatment”).

***Satisfaction with first contact (for a mental or long-term physical health condition)***

Participants who stated that they had experienced a mental or long-term physical health condition were asked to state the year they first sought help and to rate their satisfaction with the NHS response at the time.

***Satisfaction with 12 month contact (with mental or physical health services)***

All participants were asked to select all the forms of contact they had had with (1) NHS mental health services and (2) NHS physical health services in the 12 months prior to completing the survey (extended data). Participants who chose the response option “none of the above” were not asked to provide a satisfaction rating. Participants who chose one or more of the other response options (including those who selected “I have approached the NHS regarding my [mental/physical] health but have not received any treatment or support”) were asked to provide a single satisfaction rating.

***Satisfaction with 12 month treatment (mental or physical)***

In order to capture satisfaction with treatment actually received, we created a variable which removed any cases where participants had selected “I have approached the NHS regarding my [mental/physical] health but have not received any treatment or support”. Treatment for a physical health condition was examined initially as any form of physical health care, and secondly as health care for a long-term physical illness only. This was to aid comparison with mental health treatments, which are usually only provided in the presence of a mental illness. 

### Statistical analysis

All analyses were performed in IBM SPSS Statistics for Windows, Version 24. In cases where ordinal logistic regression with proportional odds was used, the assumption of proportional odds was tested using a full likelihood ratio test.

***Satisfaction with provision of NHS services***

**Satisfaction by type of health condition (mental or physical).** First, a Wilcoxon signed-ranks test was used to compare satisfaction with first NHS contact for a mental health condition vs first NHS contact for a long-term physical health condition. Participants were included in the analysis if they indicated that they had approached the NHS for both a mental health condition and a long-term physical health condition, whether or not they had actually received treatment in either case.

**Satisfaction by type of health care sought in last 12 months (mental or physical).** Next, a Wilcoxon signed-ranks test was used to examine participants' satisfaction with the NHS response when they had attempted to access mental vs physical health care in the 12 months prior to survey completion. Participants were included if they had approached the NHS for mental and physical health care, irrespective of whether they received treatment.

**Satisfaction with NHS mental health care over time.** Following this, a Wilcoxon signed-ranks test was used to examine whether there was a difference between participants’ satisfaction with the provision of NHS mental health services when they first contacted them compared to during the 12 months prior to survey completion.

***Access to NHS mental health treatment***

The percentages of treatment-seeking people who reported that they had approached the NHS and received no treatment/support were calculated for mental and physical health care. Similarly, the percentages of treatment-seeking people who indicated that they had received some form of treatment but had also been unsuccessful in accessing other treatment were calculated for mental and physical health care. A Mann Whitney-U test was used to examine whether difficulty accessing treatment reduced participants’ 12 month NHS satisfaction score.

***Satisfaction with treatment received from the NHS***

In the following analyses, satisfaction ratings from participants who stated that they asked for treatment but did not receive it (even if they also endorsed receipt of an alternative treatment) were removed. This allowed for examination of satisfaction with the treatment actually received. Wilcoxon signed-ranks tests were used to examine whether there was a difference between satisfaction with mental and physical health treatment received in the previous 12 months for all types of physical health care, and for physical health care for a long-term health condition only. Finally, satisfaction rates by type of mental health treatment were examined. 

***Satisfaction by participant mental health condition(s)***

Ordinal regression was used to examine whether the presence of specific mental health conditions were associated with higher or lower satisfaction with mental health services (relative to the average satisfaction level across conditions), either at first contact or in the last 12 months.

## Results

### Participants

Participant demographics for the whole survey sample (n = 2,187) are presented in Table 1. Respondents contain a similar proportion of people in each ethnic group as in the wider UK population ( Table 1), although men and people without higher qualifications were under-represented despite aforementioned recruitment efforts. The sample presented here covers all nations of the UK, with the most participants from England and the fewest from Northern Ireland. Participants’ ages range from 18 to 87, with a median age of 46 - slightly higher than 40, the median age of the UK population ( Office for National Statistics, 2020). Given that about 21% of individuals were believed to live with physical or mental disability in the UK at the time of the survey ( Department for Work and Pensions, 2020), it is apparent that the current sample contains more individuals with a physical disability (53%; Table 1) than the wider population. Similarly, due to deliberate over-sampling, 65% of the survey sample reported having had a mental illness at some time in their life, compared to an English population average of 26% ( Bridges, 2015).

**Table 1.** Participant demographics.

		Experience of mental illness (ever)		
	All participants	Yes	No	UK population estimate
Total, n	2187	1087 (65.09%)	529 (31.68%)	
Gender, n (%)				
Male	514 (31.28%)	314 (29.51%)	187 (35.82%)	49.1%
Female	1094 (66.59%)	721 (67.76%)	334 (63.98%)	50.9%
Non-binary or prefer to self-describe	19 (1.16%)	18 (1.69%)	0 (0.00%)	unav.
Prefer not to say	16 (0.97%)	11 (1.03%)	1 (0.19%)	n/a
Location, n (%) ^a^				
England	1050 (63.99%)	695 (65.38%)	316 (60.77%)	84.27% ^a^
Scotland	490 (29.86%)	300 (28.22%)	174 (33.46%)	8.18% ^a^
Wales	65 (3.96%)	49 (4.61%)	15 (2.88%)	4.72% ^a^
Northern Ireland	19 (1.16%)	10 (0.94%)	9 (1.73%)	2.83% ^a^
Outside UK	17 (1.04%)	9 (0.85%)	6 (1.15%)	n/a
Ethnicity, n (%)				
White	1438 (88.38%)	961 (91.26%)	431 (83.37%)	87.2%
Asian/Asian British	92 (5.65%)	38 (3.61%)	51 (9.86%)	7%
Black/African/Caribbean/Black British	53 (3.26%)	24 (2.28%)	26 (5.03%)	3%
Mixed/multiple ethnic groups	32 (1.97%)	21 (1.99%)	7 (1.35%)	2%
Other	12 (0.74%)	9 (0.85%)	2 (0.39%)	0.9%
Highest education, n (%)				
Postgraduate degree or professional qualification	587 (36.01%)	383 (36.23%)	181 (34.94%)	42% ^b^
Undergraduate degree	390 (23.93%)	260 (24.60%)	122 (23.55%)	
Vocational or college qualification	246 (15.09%)	147 (13.91%)	91 (17.57%)	21% ^b^
A-levels or equivalent	189 (11.60%)	121 (11.45%)	62 (11.97%)	
GCSEs or equivalent	200 (12.27%)	135 (12.77%)	55 (10.62%)	29% ^b^
Primary school	18 (1.10%)	11 (1.04%)	7 (1.35%)	8% ^b^
Experience of physical disability or long-term physical illness (ever), n (%)				
Yes	871 (52.56%)	598 (55.84%)	244 (46.21%)	unav.
No	774 (46.71%)	468 (43.70%)	283 (53.60%)	
Prefer not to say	12 (0.72%)	5 (0.47%)	1 (0.19%)	
Age (years), mean (SD)	45.58 (17.38)	44.44 (17.03)	48.26 (17.74)	40.3 ^a^

*Notes.* “Prefer not to say” responses to mental illness question are not presented. UK population estimates are taken from the 2011 UK Census unless otherwise stated. ^a^Estimates taken from the Office for National Statistics (2020). ^b^Estimates derived from Office for National Statistics (2017). “unav” = currently unavailable for whole UK population. n/a = not applicable.

### Satisfaction with provision of NHS services

**Figure 2.**
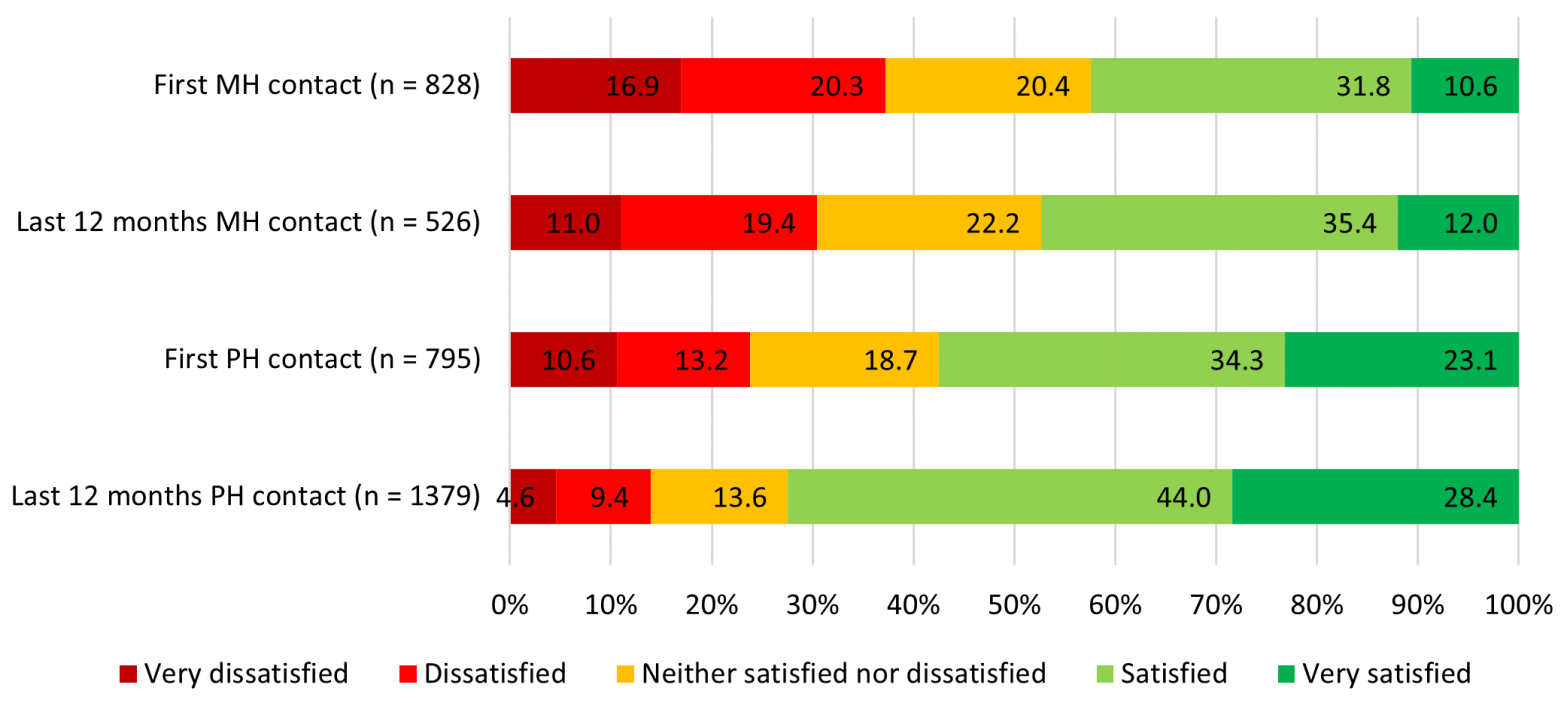
Satisfaction with mental and physical health care provision, at first contact and in the previous 12 months. MH = mental health, PH = physical health. *Note the data presented in this figure include satisfaction ratings for all NHS contact, irrespective of whether any actual treatment was provided*.

Satisfaction with NHS mental and physical health services at first contact and in the last 12 months are illustrated in Figure 2.

***Satisfaction by type of health condition (mental or physical)***

A Wilcoxon signed-ranks test amongst individuals who had both a mental health condition and a long-term physical health condition (n = 452) showed that satisfaction with first contact with physical health services was higher than satisfaction with first contact with mental health services (Z = 4.01, p < .001).

***Satisfaction by type of health care sought in last 12 months (mental or physical)***

Satisfaction was then examined amongst participants who had contacted the NHS to seek both mental and physical health care (of any kind, not specifically regarding long-term physical health conditions) during the 12 months prior to the survey (n = 452). Participants were more satisfied with the response from physical health services than the response from mental health services (Z = 7.08, p < .001).

***Satisfaction with NHS mental health care over time***

Participants were significantly more satisfied with the response from NHS mental health services during the previous 12 months than at the time they first sought these mental health services (n = 484, Z = -5.814, p < .001). There was however no significant relationship between satisfaction at first contact and years since first contact (r(825) = -0.05, p = 0.12).

### Access to NHS mental health treatment

During the 12 months prior to the survey, 526 respondents had sought mental health care and 1379 had sought physical health care (this included all forms of physical health care, not just care related to long-term physical health conditions). The percentage of people who reported that they had approached the NHS and received no treatment/support was 11.4% (n = 60) for mental health care and 4.6% (n = 63) for physical health care. An additional 8.0% (n = 42) for mental health care and 9.1% (n = 126) for physical health care had only received some of the treatment/support they sought.

A Mann Whitney-U test showed that participants who received the mental health treatment they asked for (n = 424) were more satisfied with the NHS than those who did not receive all the mental health treatment they asked for (n = 102, Z = - 8.81, p < .001). Similarly, participants who received the physical health treatment they asked for (n = 1190) were more satisfied than those who did not receive all the physical health treatment they asked for (n = 189, Z = - 11.43, p < .001).

### Satisfaction with treatment received from the NHS

**Figure 3.**
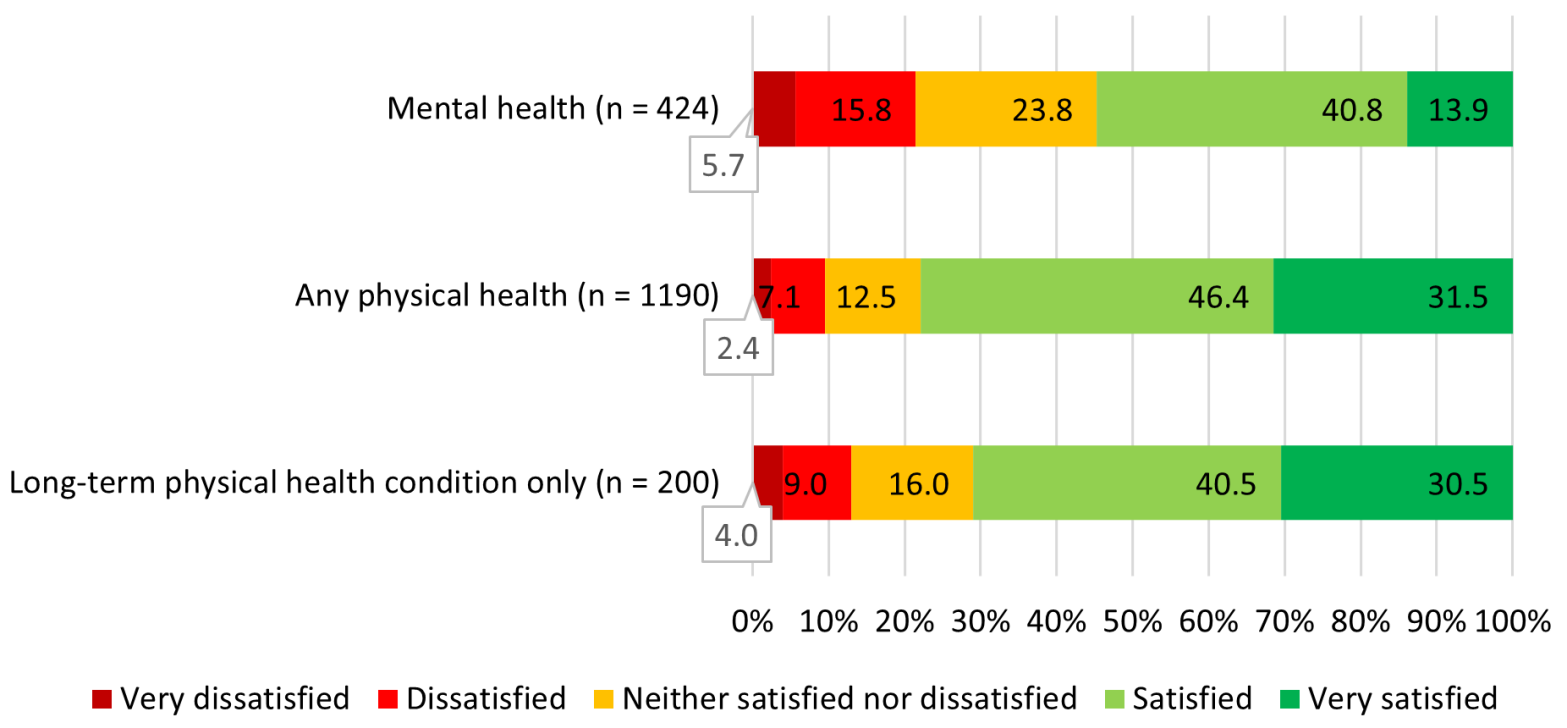
Satisfaction with treatment received in the previous 12 months. *“Mental health” = satisfaction scores for all participants who received the treatment they requested (n = 424), “Any physical health” = satisfaction scores for all participants who received the physical health treatment they requested (n = 1190), “Long-term physical health condition only” = satisfaction scores for participants whose only physical health treatment was in relation to a long-term physical health condition (n = 200)*.

Satisfaction with NHS mental and physical health treatment actually received in the previous 12 months is presented in Figure 3. A Wilcoxon signed-ranks test was used to examine whether there was a difference between satisfaction with mental and physical health treatment received in the previous 12 months. It was found that participants who had received both forms of health care (n = 324) were significantly more satisfied with the treatment for physical health than the treatment for mental health (Z = 5.71, p < .001). However, there was no difference between satisfaction with treatment for mental vs long-term physical health conditions amongst participants who received both (Z = 0.90, n = 47, p = .37).

**Figure 4.**
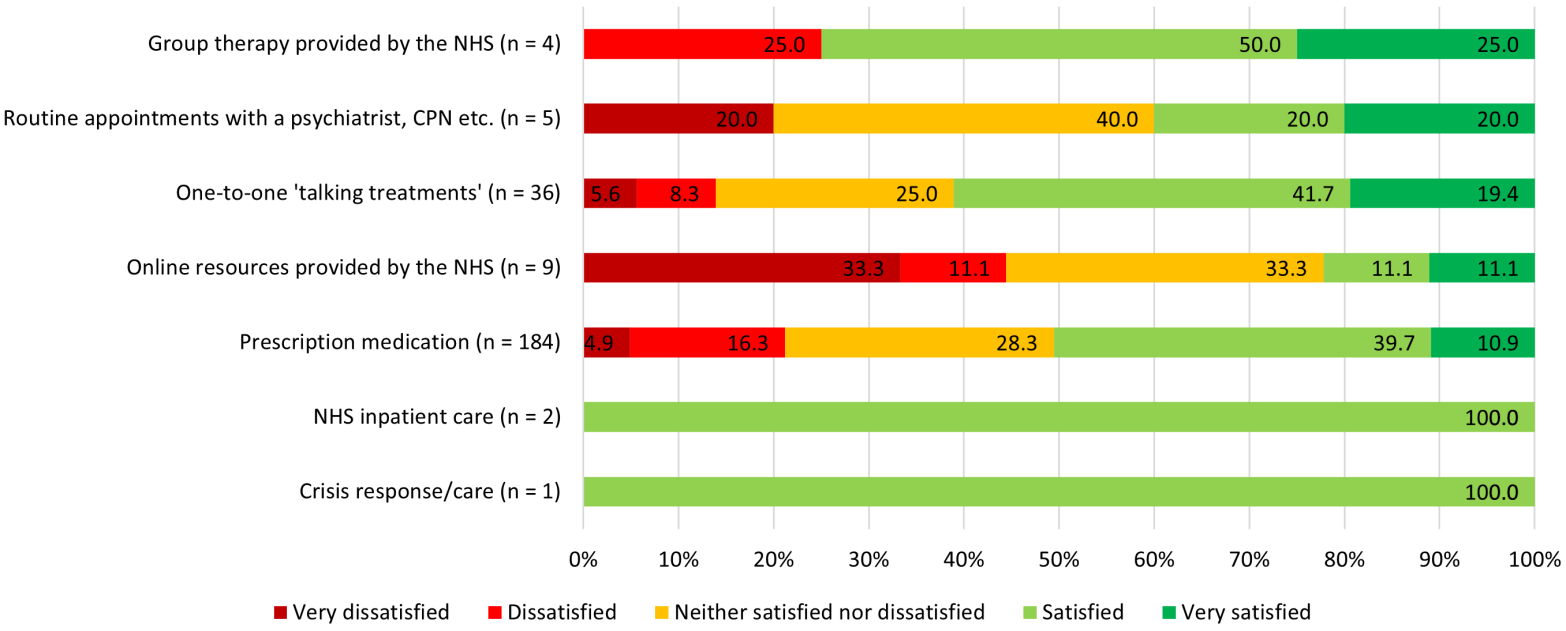
Satisfaction scores by treatment type (n = 241). Participants were included if they had received only one treatment type in the 12 months prior to completing the survey.

When examining satisfaction scores by treatment type (in the n = 241 participants who stated that they had received only one treatment type in the 12 months prior to completing the survey), 50.5% of those (n = 189) who had received prescribed medication were very/satisfied, and a further 28.3% neutral, as compared to 61.1% (and neutral 25.0%) of those who received one-to-one talking treatment (n = 36). See Figure 4 for breakdown of satisfaction by treatment type.

***Satisfaction by mental health condition***

**Figure 5.**
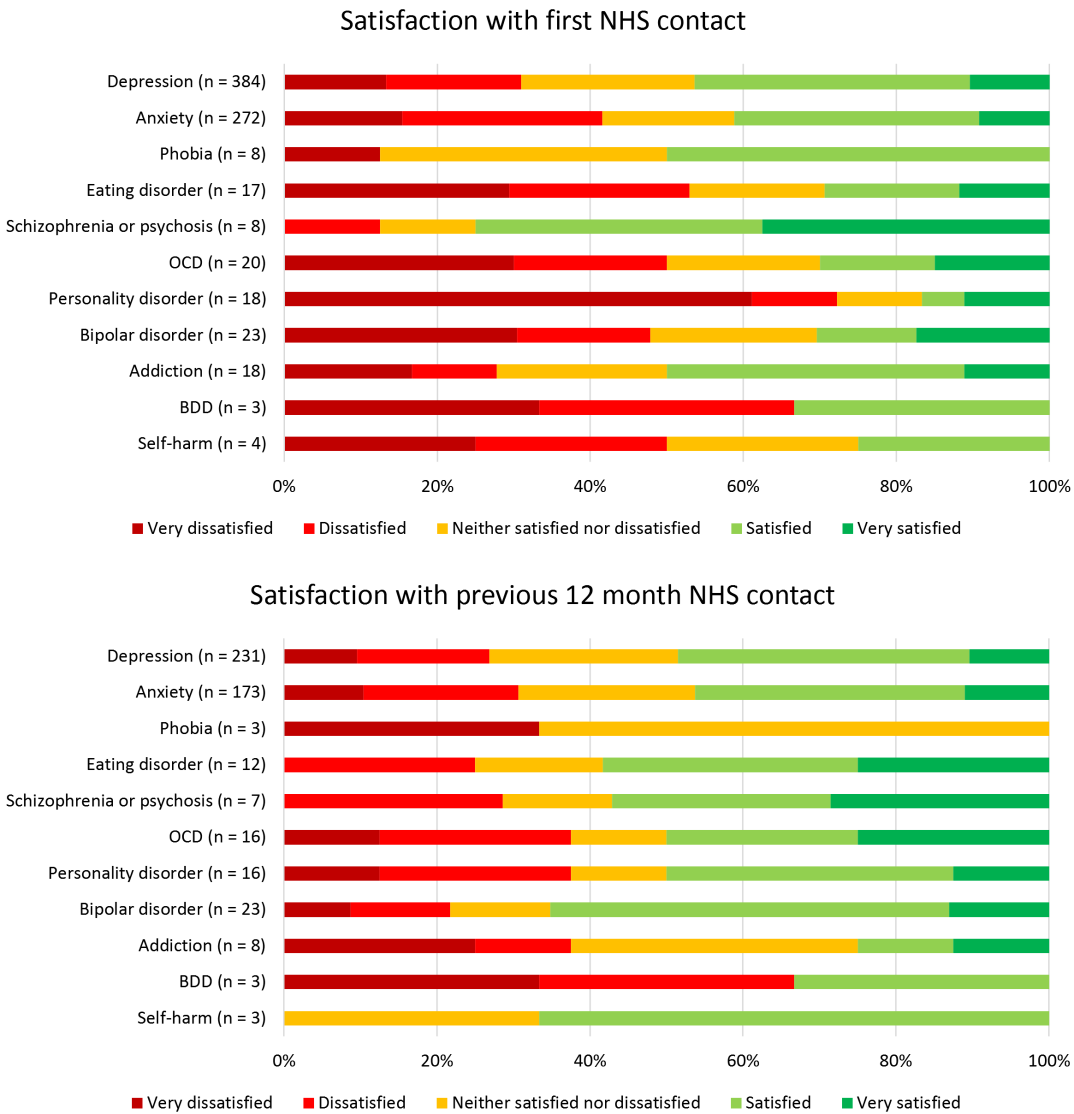
Satisfaction with NHS mental health services by primary mental illness, at first contact and during the previous 12 months. OCD = obsessive-compulsive disorder, BDD = body dysmorphic disorder. *Primary mental illness was defined as the condition that had the biggest self-reported impact on each participant’s life.*

Finally we examined the relationship between mental health condition and satisfaction with NHS contact ( Figure 5).

Ordinal regression was used to examine the relationships between mental health conditions and satisfaction with first NHS contact ( Table 2). For each participant, all measured mental health conditions were coded as present or absent, to allow for multiple conditions per person. The full ordinal regression model significantly predicted satisfaction when first seeking NHS mental health care (χ ^2^(11) = 85.68, p < .001), and the assumption of proportional odds was met (χ ^2^(33) = 29.85, p = .63). Five mental health conditions were significantly associated with lower satisfaction when first seeking NHS mental health care: depression, anxiety, eating disorder, personality disorder, and self-harm. This means that having experience of depression, anxiety, eating disorders, personality disorders or self-harm was associated with a reduction in satisfaction at first contact, relative to the average satisfaction score for participants with mental health conditions. No mental health conditions were associated with higher satisfaction.

**Table 2.** Associations between presence of mental health conditions and satisfaction with first NHS contact.

		Satisfaction with first NHS contact		
	n(present)	OR	95% CI	p value
Mental illness present				
Depression	652	0.74	0.54 to 1.00	0.047 *
Anxiety	587	0.64	0.49 to 0.86	0.002 *
Phobia	102	1.06	0.72 to 1.57	0.76
Eating disorder	124	0.55	0.38 to 0.81	0.002 *
Schizophrenia or psychosis	30	1.42	0.72 to 2.80	0.31
Obsessive-compulsive disorder	81	0.68	0.44 to 1.04	0.08
Personality disorder	49	0.36	0.20 to 0.63	< .001 *
Bipolar disorder	37	0.74	0.40 to 1.37	0.34
Addiction or substance use disorder	66	1.00	0.63 to 1.60	0.99
Body dysmorphic disorder	41	1.22	0.66 to 2.25	0.52
Self-harm	171	0.58	0.41 to 0.81	0.002 *

*Note.* n = 828; n(present) indicates number of individuals for whom a specific mental health condition was present, relative to absent. The total n(present) is greater than the number of participants because many participants had more than one mental health condition. Satisfaction with first NHS contact refers to first NHS contact for primary mental health condition. OR = odds ratio, CI = confidence interval, * = p < .05.

The same ordinal logistic regression model was then used to examine the relationship between the presence of each mental health condition and satisfaction with NHS mental health care provision over the *last 12 months* ( Table 3). The full model significantly predicted satisfaction with mental health services in the last 12 months (χ ^2^(11) = 31.13, p = .001), and the assumption of proportional odds was met χ ^2^(33) = 25.31, p = .83. The only condition associated with reduced rates of satisfaction in the last 12 months was obsessive-compulsive disorder (OCD). No mental health conditions were associated with higher satisfaction.

**Table 3.** Associations between presence of mental health conditions and satisfaction with NHS contact in last 12 months.

		Satisfaction with NHS contact in last 12 months		
	n(present)	OR	95% CI	p value
Mental illness present				
Depression	439	0.77	0.50 to 1.17	0.22
Anxiety	422	0.75	0.50 to 1.13	0.17
Phobia	82	0.75	0.48 to 1.17	0.21
Eating disorder	104	0.67	0.43 to 1.04	0.07
Schizophrenia or psychosis	28	0.75	0.37 to 1.53	0.44
Obsessive-compulsive disorder	81	0.63	0.40 to 0.98	0.04 *
Personality disorder	44	0.60	0.34 to 1.07	0.08
Bipolar disorder	37	1.55	0.83 to 2.88	0.17
Addiction or substance use disorder	52	1.01	0.60 to 1.71	0.97
Body dysmorphic disorder	38	0.93	0.48 to 1.80	0.84
Self-harm	153	0.87	0.60 to 1.27	0.48

Note: n = 526; n(present) indicates number of individuals for whom a specific mental health condition was present, relative to absent. The total n(present) is greater than the total number of participants because many participants had more than one mental health condition. OR = odds ratio, CI = confidence interval, * = p < .05.

## Discussion

This study describes satisfaction with mental and physical health services provided by the NHS across the UK. Despite nearly a decade of stated political intentions to provide parity of esteem for mental and physical health care (e.g. Health and Social Care Act, 2012), our findings suggest that access to, and satisfaction with, NHS mental health services remain lower than NHS physical health services. At the same time, a small majority of individuals stated that they were satisfied with the mental health treatment they had received in the previous 12 months, and people with mental illness were significantly more satisfied with the NHS response provided in the previous 12 months than with the NHS response provided when they had first sought help. It is unclear whether this indicates improvement in service provision over time, or greater satisfaction as a patient moves along their treatment pathway. Further, when examining services received in the previous 12 months, there was no significant difference between satisfaction with mental health services and services for long-term physical illness.

The present findings broadly align with Appleby *et al.*’s (2020) report which found that, in 2019, 60% of the public were satisfied with the NHS as a whole. Similarly, our observation that satisfaction with physical health services was higher than with mental health services accords with the findings of the most recent comparable (pre-COVID-19 pandemic) NHS Patient Surveys. In these surveys, the percentage of people who selected the highest “experience” option (corresponding to “I had a very good experience”) was somewhat lower in the 2019 survey that focused on mental health (community mental health services; 17%; Care Quality Commission, 2020b) than in the 2019 survey on adult inpatient services (27%; Care Quality Commission, 2019) or the 2018 survey on accident and emergency services (29%; Care Quality Commission, 2018).

Though the use of medication for mental health has been criticised in some quarters ( Gardner & Kleinman, 2019), the majority (50.5%) of participants who had received medication (and had not requested or received any other mental health treatment) in the previous 12 months were satisfied or very satisfied with this treatment, while only 21.2% were (very) dissatisfied. This finding is largely in agreement with the 2019 community mental health survey, which found that 87% of respondents felt that taking medicine had helped their mental health ( Care Quality Commission, 2020a).

Whilst many public health campaigns focus on the need to “ask for help”, it is important to note that in the present research, 1 in 10 people who did ask for help were unable to access it at all, with a further 8% unable to access all the help they requested. Notably, the proportion of people unable to access requested mental health care (11.4%) was double the proportion of people unable to access requested physical health care (4.6%), despite higher absolute numbers of people seeking physical health care. The fact that satisfaction was significantly higher amongst those who were able to access all the health care they requested suggests that some of the public dissatisfaction with mental health care is based on difficulties gaining access to it.

It was found that the presence of depression, anxiety, eating disorder, personality disorder and self-harm were associated with lower satisfaction when an individual first contacted NHS mental health services. Eating disorders and personality disorders have both been identified as requiring additional attention within the NHS ( Dale *et al.*, 2017; Parliamentary and Health Service Ombudsman, 2017), whilst self-harm and borderline personality disorder have previously been associated with stigma from mental health professionals ( Henderson *et al.*, 2014). The presence of lower satisfaction associated with anxiety and depression is less expected, though the fact that this effect is not present in satisfaction ratings for the previous 12 months is encouraging. However, the absence of a significant association between year of first contact with mental health services and satisfaction with initial contact suggests that rates of satisfaction may not have increased in a linear fashion over time. The finding that none of the aforementioned mental health conditions were associated with lower NHS satisfaction in the last 12 months could be indicative of improvement in the parity of NHS response across mental health conditions. With respect to the previous 12 months, only people with OCD reported lower levels of satisfaction with NHS health services. This suggests that public satisfaction with NHS mental health services would benefit from additional focus on OCD and its treatment. 

One of the strengths of this study is its demographically diverse sample, with the ethnicity and age of those taking part reflecting the broader make-up of the UK. Similarly, participants were recruited from across the four UK nations, and people with a physical disability or long-term health condition were well represented. Nevertheless, despite recruitment efforts, there were more female than male participants, and the sample as a whole reported higher levels of education than the general UK population. The study is also limited by the use of self-report; it is not possible to confirm whether those taking part in the survey had a diagnosis of the mental health conditions reported, nor whether they had actually accessed NHS health services. Despite this, satisfaction with NHS services is more widely represented in the present study than in research which focuses only upon people already in receipt of health care, such as the Care Quality Commission’s NHS Patient Surveys.

The present study offers a valuable snapshot of satisfaction with NHS mental health services in 2019, which just happened to be the year prior to the emergence of the Covid-19 pandemic. Future work should build on this by examining how satisfaction has changed since the pandemic began. This would add valuable evidence to the ongoing discussion surrounding the ways that the pandemic and consequent lockdowns have impacted both mental health itself and NHS mental health care ( Pan *et al.*, 2021). Furthermore, it would be useful to examine whether online services (which had the lowest satisfaction ratings amongst the n = 9 who received this single treatment type), have improved following the move away from face-to-face healthcare during the pandemic. It is possible that increased use of these services in recent years has generated service improvements and also normalised this kind of service delivery, both of which could have a positive impact on satisfaction ratings. Future research would also benefit from measuring satisfaction at different time points; whilst the present study examined satisfaction with first contact and recent (previous 12 month) contact, both scores were taken at the same time and could have been subject to biases in memory.

In conclusion, we found that satisfaction with NHS mental health services was lower than satisfaction with NHS physical health services in general, though satisfaction rates were similar when comparing mental health services to services for long-term physical health conditions. There was some evidence of improvement in satisfaction with mental health services from first to most recent contact, as well improved parity in the level of satisfaction across different mental health conditions. Satisfaction ratings for the 12 months prior to the survey (conducted before the COVID-19 pandemic) indicated relatively similar levels of satisfaction across different types of mental health treatment, including medication and talking treatments. Future work should build upon these findings to examine how satisfaction with health services has changed in the wake of the COVID-19 pandemic.

## Data availability

### Underlying data

Edinburgh DataShare: Views on sharing mental and physical health data among people with and without experience of mental illness. https://doi.org/10.7488/ds/3486 ( Kirkham *et al.*, 2022b)

This project contains the following underlying data files:

- Dataset_Views_on_sharing_health_data.xlsx

### Extended data

Edinburgh DataShare: Views on sharing mental and physical health data among people with and without experience of mental illness. https://doi.org/10.7488/ds/3486 ( Kirkham *et al.*, 2022b)

This project contains the following extended data files:

- Data_dictionary.xlsx
- Dataset_files.txt
- Open access version of survey.pdf
- License_text.txt

Data are available under the terms of the Creative Commons Attribution 4.0 International license (CC-BY 4.0).
