# Supplementary material for: Patient satisfaction with mental and physical health services: Findings from a UK-wide online survey
Source: Wellcome Open Res. 2022 Aug 1;7:198. [Version 1] doi: 10.12688/wellcomeopenres.17973.1 (PMC10480769; doi:10.12688/wellcomeopenres.17973.1)
Supplement: Peer review history

# Peer review: Reviewer response for version 1

**DOI:** 10.21956/wellcomeopenres.19923.r62240

**Published:** 2023-09-06

**Authors:** Jacek Moskalewicz

**Affiliations:** 1 Department of Studies on Alcohol and Drug Dependence, Institute of Psychiatry and Neurology, Warsaw, Poland

1. Important topics
  1. involving patients in evaluation of the treatment they receive,
  2. comparing satisfaction from first and most recent contact
  3. treatment of mental and physical  conditions, including long term disabilities or physical illness.
2. Results, which complement routinely collected data, should promote further public debate to improve quality of NHS services, in particular mental health services and satisfaction of their clients.
3. Participatory elaboration of research tools including stakeholders such as service users and clinicians should be acknowledged.
4. Genuine efforts to recruit representative sample of NHS users with conscious decision to oversample people with mental health conditions. Nevertheless, it would be useful to learn how many respondents were recruited by different recruitment approaches, and what were refusal rates.
5. It is difficult to assess how successful these efforts were based on table 1. In its last column UK population estimates are provided but it is not clear if these estimates refer to UK general population or UK general population aged 16+ to be comparable with the study sample. 
6. Moreover, there seems to be a huge percentage of missing data or “prefer not to say” responses as numbers of male, female, non-binary and “prefer not to say” sum up to 1643 while a total number of participants was 2187. If that difference which constitutes 25% of the whole sample or 544 respondents, who refused to confirm their experience with mental illness and relevant services then those respondents deserve more detailed description as their refusals may be source of significant bias.
7. There are further inconsistencies which deserve more detailed explanation e.g. from figure 5 we learn that there were 8 people with phobia who expressed their level of satisfaction with NHS first contact while from table 2 we realised that there were 102 respondents with phobia who were included in regression showing association between presence of mental health condition and satisfaction with first NHS contact.
8. Most of the mental health conditions presented in figures 5 and 6 are very small, except for depression and anxiety disorders, respectively 384 and 272 while all remaining range from 3 to 23. Therefore, I would suggest reduction to three categories: depression, anxiety and other psychiatric conditions.

Is the work clearly and accurately presented and does it cite the current literature?

Partly

If applicable, is the statistical analysis and its interpretation appropriate?

Partly

Are all the source data underlying the results available to ensure full reproducibility?

Partly

Is the study design appropriate and is the work technically sound?

Partly

Are the conclusions drawn adequately supported by the results?

Partly

Are sufficient details of methods and analysis provided to allow replication by others?

Partly

Reviewer Expertise:

Sociology, epidemiology

I confirm that I have read this submission and believe that I have an appropriate level of expertise to confirm that it is of an acceptable scientific standard, however I have significant reservations, as outlined above.

# Peer review: Reviewer response for version 1

**DOI:** 10.21956/wellcomeopenres.19923.r64675

**Published:** 2023-09-05

**Authors:** David Giménez-Díez

**Affiliations:** 1 Nursing Department, Universitat Autonoma de Barcelona, Barcelona, Catalonia, Spain

The introduction is well formulated. It poses the research problem well and its importance is well justified. However, I think that the following objectives would be better if they were in the methods section:

- participants' satisfaction with the NHS response when they first sought mental vs. physical health care
- participants' access to and satisfaction with their recent NHS treatment for mental vs. physical health conditions
- change in satisfaction with NHS mental health services over time
- satisfaction with NHS mental health services by type of treatment and mental health condition.

It would be good to comment that an ad hoc questionnaire - survey was carried out because there was none validated.

 The presentation of the results is very visual and easy to read, I congratulate the authors.

 In the limitations I would include that some participants received money for their participation and others did not, which perhaps could have motivated the participation of some more.

 It is an interesting article, especially since it was carried out just before the appearance of COVID-19, and its comparison with current levels of satisfaction could provide decisive data for the health system.

 With a few changes I recommend its indexing.

Is the work clearly and accurately presented and does it cite the current literature?

Yes

If applicable, is the statistical analysis and its interpretation appropriate?

I cannot comment. A qualified statistician is required.

Are all the source data underlying the results available to ensure full reproducibility?

Yes

Is the study design appropriate and is the work technically sound?

Yes

Are the conclusions drawn adequately supported by the results?

Yes

Are sufficient details of methods and analysis provided to allow replication by others?

Yes

Reviewer Expertise:

Mental Health Nursing, Crisis Resolution Home Treatment Teams, Mental Health Nursing and Psychotherapy, Nursing and Gender

I confirm that I have read this submission and believe that I have an appropriate level of expertise to confirm that it is of an acceptable scientific standard.

# Peer review: Reviewer response for version 1

**DOI:** 10.21956/wellcomeopenres.19923.r51792

**Published:** 2022-08-15

**Authors:** Debra Rickwood

**Affiliations:** 1 Faculty of Health, University of Canberra, Canberra, ACT, Australia

This article reports the findings of an online survey of patient satisfaction with mental and physical health NHS services. The survey was conducted from December 2018 to August 2019, with participants recruited via social media, posters, and in-person, and additional recruitment attempts to increase participation from some under-represented demographic groups. The findings from four questions about satisfaction with NHS services showed that satisfaction was substantially less for mental health compared with physical health services at both first contact and in the prior 12 months. For both health conditions, more recent satisfaction was higher.

 The paper is very well written and clearly presented and addresses an issue that needs to be monitored, which has not occurred sufficiently to date. The methods are well described and the results clearly presented in text and figures. The discussion raises important issues and implications.

 My only concerns were the need for some further consideration of limitations. It is noted that there was no relationship between satisfaction at first contact and years since contact, however, details are not provided for years since contact. It would be of interest to know how far back in time first contact was for participants. Presumably, there is considerable variability in the timing of first contact, and this would be confounded with age. The reliability and validity of the first contact-related satisfaction measures are likely compromised, and this should be noted as a limitation as well as providing more information on this measure.

 Another limitation is that some of the results are based on very small numbers. For example, in Figure 4, the n’s are very small for all except prescribed medication. Similarly, Figure 5 shows very small numbers for many mental health conditions. It is not appropriate to draw any conclusions from such small numbers, for example, from four people reporting self-harm.

 Finally, the interpretation of the results for medication use seems overly positive. Stating that “only 21.2% were (very) dissatisfied” does not seem to warrant the qualification of ‘only’; having over one-fifth dissatisfied is quite substantial. Further, it does not seem to be justified to claim that results showing 50.5% were satisfied are similar to findings from the Care Quality Commission report that 87% of respondents felt that taking medicine had helped their mental health.

Is the work clearly and accurately presented and does it cite the current literature?

Yes

If applicable, is the statistical analysis and its interpretation appropriate?

Yes

Are all the source data underlying the results available to ensure full reproducibility?

Yes

Is the study design appropriate and is the work technically sound?

Yes

Are the conclusions drawn adequately supported by the results?

Yes

Are sufficient details of methods and analysis provided to allow replication by others?

Yes

Reviewer Expertise:

My expertise is mental health services research and evaluation. I have expertise in service satisfaction measures.

I confirm that I have read this submission and believe that I have an appropriate level of expertise to confirm that it is of an acceptable scientific standard.
